# A Rare Case of Occult Extra-Baudet Period in a Patient With Spontaneous Splenic Rupture

**DOI:** 10.7759/cureus.16707

**Published:** 2021-07-29

**Authors:** Snehasis Das, Oseen Shaikh, Naveen Kumar Gaur, Chellappa Vijayakumar, Uday Kumbhar

**Affiliations:** 1 Surgery, Jawaharlal Institute of Postgraduate Medical Education and Research, Puducherry, IND

**Keywords:** splenic rupture, baudet period, blunt trauma, splenic hematoma, splenectomy

## Abstract

Spontaneous splenic rupture in the extra-Baudet period is a rare phenomenon demanding a high clinical suspicion for diagnosis with prompt treatment. We present a case of a 39-year-old male presenting with left upper abdominal pain following six months after abdominal trauma. The patient underwent imaging studies showing a large subcapsular splenic hematoma with near-total parenchymal displacement and moderate hemoperitoneum. Sequential scans revealed a non-progressive resolving hematoma with no active extravasation. The patient underwent aggressive initial resuscitation followed by successful conservative management and was discharged.

## Introduction

Spontaneous splenic rupture (SSR) is postulated to be only one percent of all cases of splenic ruptures [1]. Potentially life-threatening, splenic ruptures are to be diagnosed promptly and be given immediate attention, for which clinicians need to have a high suspicion index. Very few cases have been reported, mostly presenting following a traumatic event and few as delayed hemorrhage in the period of Baudet amounting to a maximum of one week [2]. Depending on the extent of injury, splenic involvement, and clinical status of the patient, treatment lines are kept flexible. We present a 39-year-old male, diagnosed with spontaneous extra-Baudet period splenic rupture, managed conservatively, and discharged with no further complications.

## Case presentation

A 39-year-old male with no co-morbidities presented with complaints of upper abdominal pain with on and off vomiting for one week. The pain was mostly centered around the epigastrium and the left hypochondrium, mild to moderate in severity, non-radiating, relieving with analgesic medications. The patient had occasional vomiting non-bilious for one week, not associated with food intake. The patient lost appetite and significant weight over the last one month. The patient had a history of blunt trauma abdomen six months back where a bullock cart had rolled over his abdomen on the left side. He has been asymptomatic and was managed conservatively at a local hospital. On examination, the patient had pallor, was mildly dehydrated, and vitals were stable. There was mild tenderness in the left hypochondrium, with voluntary guarding but no evidence of rigidity and normal bowel sounds on auscultation.

**Figure 1:**
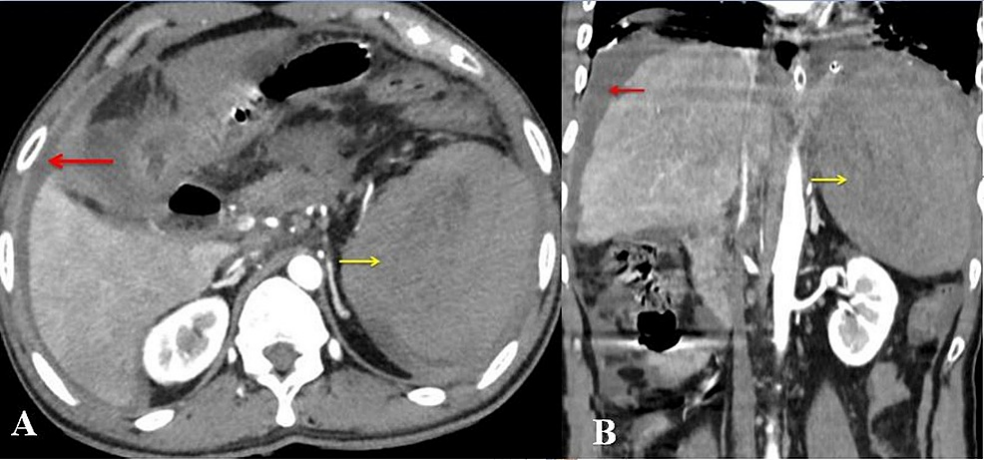
Computed tomography showing arterial phase showing peri-splenic hematoma (yellow arrow) and free fluid abdomen (red arrow). (A) Axial view. (B) Coronal view.

Blood investigations showed mild anemia (10.2 g/dL) and leukocytosis. The liver function test (LFT) and renal function test (RFT) were normal. Amylase and lipase were normal. Chest x-ray of the patient revealed a minimal left-sided costophrenic angle blunting with no other abnormalities. The abdomen's ultrasound showed an 8.5 cm x 5.2 cm wide heterogeneous hyperechoic subcapsular collection with no internal vascularity or moving echoes possibility of a splenic subcapsular hematoma. Contrast-enhanced computed tomography (CT) abdomen and thorax showed a large splenic hematoma measuring 10 cm x 8 cm, replacing the entire parenchyma with normal splenic vessels and no active extravasation, with free fluid in the abdomen suggestive of delayed splenic rupture (Figure 1).

**Figure 2:**
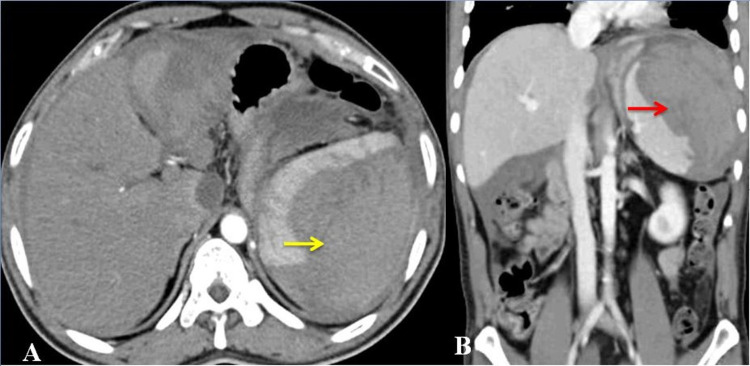
Computed tomography showing (A) arterial phase showing peri-splenic hematoma (yellow arrow) (axial view) and (B) venous phase showing peri-splenic hematoma (red arrow) (coronal view).

The patient was planned for a conservative line of management with abdominal girth measuring and serial hemoglobin monitoring with a need-based blood transfusion. In the initial two days, the patient's hemoglobin demonstrated a decreasing trend with a drop from 10.2 g/dL to 6.4 g/dL within 48 hours, for which the patient was given three packed red blood cell transfusions. After three days, the patient's hemoglobin started picking up, and counts were stabilized. There was a marked improvement in the abdominal signs following which the patient was started on orals, and he tolerated it well. CT abdomen was repeated after one week to detect any active source of bleed or an expanding hematoma. There was no evidence of any active extravasations of the contrast or expansion of the hematoma, reducing the free fluid in the abdomen (Figure 2).

The patient was symptomatically better and was planned for discharge. The patient was followed up for the next three months with no further complications.

## Discussion

SSR is a rare phenomenon to envisage in the medical realm. Due to its mysterious form of presentation, it is potentially a life-threatening situation most of the time. Usually, it is seen because of neoplastic, infectious, hematological, inflammatory, or other metabolic causes that precede such a clinical entity. Knobloch differentiated non-traumatic rupture of a pathological spleen from the rare non-traumatic splenic rupture of unknown etiology; an actual SSR [2,3].

The kindred mortality with splenic rupture demands a high degree of suspicion, rapid diagnosis, and prompt intervention to mitigate its lethality. Typically, in an emergency, most cases present with a clinical spectrum of hemorrhagic shock with massive intraperitoneal bleed requiring an immediate splenectomy. Significantly few present in the form of a delayed rupture with a massive bleed of more than 500 mL nearly days to a week after the injury, described as the period of Baudet. Less than one percent of the patients seems to be the true SSR that happens out of the blue in the background of no established medical predicament [1]. Our patient had presented six months later to trauma and had crossed the Baudet period, a rare scenario that has never been reported.

Many hypotheses have been postulated regarding the causality of SSR, which includes the theory of expanding hematoma pressure to the developing intraparenchymal venous congestion resulting in occult rupture. However, none have come to any valid conclusions. Orloff and Peskin formulated a set of criteria that would define an SSR. These include the absence of a history of trauma or unusual effort that could injure the spleen, the absence of peri-splenic adhesions suggestive of previous trauma, the absence of preexisting splenic disease, and normal microscopic and macroscopic appearance of the spleen. Later on, even the fifth criterion was added by Crate and Payne, which reads the acute phase and convalescent sera studies should show no rise in viral antibody titers suggestive of recent infection with types associated with splenic involvement. Our case obliges to all the discussed criteria in addition to being one of a kind in an occurring way out of the proposed period of Baudet for delayed splenic ruptures.

A watch out for the classic signs of left upper abdominal pain with guarding and hemodynamic instability must be kept in mind in the absence of any trauma. In addition, a sharp left shoulder tip pain from diaphragmatic irritation, also known as Kehr's sign, and a palpable tender left hypochondrial mass known as the Balance sign might aid in the diagnosis. In our case, the absence of all the above-mentioned ominous signs made the diagnosis difficult. Another set of differentials that should be kept in mind to alert against any masquerader are cardiac ischemia, pulmonary embolism, left lobe pneumonia, peptic ulceration, or ruptured sigmoid diverticulitis [4].

CT scan of the abdomen with the thorax is necessary to seal the deal in diagnosing the condition with proper grading of severity of the splenic injury. The most common findings would tilt toward splenomegaly with or without splenic laceration with an intraparenchymal or subcapsular bleed. Based on the CT findings, splenic injury can be graded into five categories [5].

On the scale described above, low-grade injuries are usually managed conservatively, whereas the higher-grade injuries demand surgical exploration based on clinical presentation [6]. However, evidence has suggested that the patient's clinical condition rather than radiology and pathology decide the modality of treatment. But even after so, each patient has to be dealt with individually with a low threshold for surgical intervention in case of hemodynamic collapse. In our case, the patient was given a trial of conservative management, which heralded signs of recovery, and was discharged successfully without any need for splenectomy. Post-discharge, the patient was followed up for three months, and there were no signs suggestive of any hemodynamic compromise.

## Conclusions

A true SSR is a rare element in the medical Eminem and could veil a potentially life-threatening clinical outcome. It requires a high degree of suspicion for diagnosis. The severity can be misjudged and need close monitoring based on hemodynamic status to decide between a non-surgical versus a surgical treatment approach. Lower degree injuries can be given a trial of conservation before the acclamation for a surgical endpoint, whereas higher degree injuries warranting an immediate splenectomy. Although not described in the literature, splenic ruptures beyond the period of Baudet do exist as in our case and require an accurate hawk eye intuition to diagnose such a rare pestilence.
